# Experimental Research on and Optimization of Plasma Emitter Sources

**DOI:** 10.3390/s25061715

**Published:** 2025-03-10

**Authors:** Xu Gao, Jing Zhou, Xiao Du

**Affiliations:** 1 College of Petroleum Engineering, Xi’an Shiyou University, Xi’an 710065, China; 20111010002@stumail.xsyu.edu.cn (X.G.); 22111010007@stumail.xsyu.edu.cn (X.D.); 2 National Engineering Laboratory for Oil and Gas Drilling Technology, Xi’an Shiyou University, Xi’an 710065, China

**Keywords:** needle–plate structure, impulse wave, plasma emitter

## Abstract

Traditional emitters used for downhole acoustic detection have limited radiation frequency and energy, making it difficult to transmit high-precision acoustic signals over long distances. This paper presents a plasma emitter in which high-pressure discharge generates a powerful spherical impulse wave with a wide frequency range. First, the discharge characteristics of the plasma needle-plate emitter are analyzed using high-voltage discharge experiments and discharge simulation models for underwater emitters. Subsequently, advanced modifications are made to the structure of the needle–plate emitter to meet the requirements of downhole detection. A new type of hollow needle–plate emitter with a spherical tip is developed. The results show that the structural optimization of the hollow needle–plate emitter with a spherical tip resulted in a 27.2% increase in impulse wave amplitude, a 28.1% improvement in electro-acoustic conversion efficiency, and a radiation frequency band covering up to 100 kHz. This development is conducive to more accurate and longer-range downhole structure detection. The detection range outside the borehole can reach tens to hundreds of meters. This enables the precise control of the wellbore path and reduces the demands on the rig’s build rate. The emitter has significant application potential in areas such as onshore and offshore oil and gas exploration, unconventional resource detection, impulse wave fracturing and wellbore clearance, and rescue and U-well drilling.

## 1. Introduction

As fields enter later stages of development, the development targets shift from medium-to-shallow structural oil and gas reservoirs to deep, tight, and complex small fault-block reservoirs. The focus of reservoir description shifts from structural traps and large faults to thin interlayers, fracture-type reservoirs, and the distribution of residual oil. The target reservoirs are deeper and smaller and have more complex reservoir characteristics [1]. At the same time, the focus of development is shifting from conventional to unconventional reservoirs [2]. The exploitation of unconventional reservoirs presents significant challenges, including higher development costs and the demand for economic benefits and high recovery factors. This has led to increased requirements for detailed reservoir characterization [3]. Finding an emitter that enables long-distance acoustic transmission and high-precision characterization has always been a relentless pursuit in acoustic detection technology [4,5,6].

Acoustic detection technology triggers acoustic signals in the borehole, with most of the acoustic energy traveling along the borehole wall and a small proportion traveling through the wall into the formation [7]. Increasing the energy emitted by the transmitting source allows more acoustic energy to flow deeper into the formation. When the acoustic energy encounters the geological structure, it is reflected back to the wellbore, providing long-range acoustic transmission and oil and gas detection. Increasing the radiation frequency of the emitting source results in shorter wavelengths flowing into the formation, providing better resolution for geological formation detection. Increasing the energy conversion efficiency of the emitting source can further enhance the radiated energy by reducing energy leakage and saving energy costs [8].

The development of emitter technology has led to significant advancements and evolutions in acoustic wave detection technology. The primary emitters used in downhole acoustic detection are piezoelectric sources. The earliest research focused on monopole sources, with corresponding logging instruments developed [9,10]. The radiation frequency of monopole sources is concentrated in the 10 kHz to 15 kHz range [11]. During acoustic wave propagation through the formation, the radiation energy undergoes significant attenuation, preventing long-distance detection. The sound field of the monopole source radiates in all directions, expanding spherically, and lacks directional capabilities [12]. The radiation frequency of dipole sources is concentrated below 5 kHz. These sources allow for deep acoustic wave propagation and can detect geological structures tens of meters away [13,14]. Dipole sources have some directional resolution capabilities for external reflectors, although they face the challenge of 180° ambiguity in reflector orientation [15]. Phased-array sources are based on monopole sources and are formed by arranging multiple monopole sources in a circular or linear configuration, providing more directional radiation capabilities [16]. The radiation frequency of phased-array sources is around 14 kHz. However, the fixed radiation frequency and limited transmitting power limit the ability of phased-array sources to detect geological features up to tens of meters away [17,18]. In recent years, multipole sources, which can combine the characteristics of monopole, dipole and quadrupole sources, have become a focus of research [19,20]. The radiation frequency of multipole sources ranges from 15 kHz to 25 kHz, allowing the detection of geological features within tens of meters of the borehole [21]. However, multipole sources suffer from low radiation energy and are easily disturbed by noise, such as drill string waves, which can obscure the acoustic signals carrying geological information [22].

Downhole acoustic detection typically employs conventional piezoelectric transmitters, including monopole, dipole, multipole, and phased-array sources [23,24]. These transmitters generate specific radiation frequencies through the vibration of piezoelectric ceramics of varying sizes [25]. Once manufactured, the frequency of the transmitter cannot be adjusted. Due to the limited instrument space available in downhole environments, the size of piezoelectric transmitters is constrained, which in turn restricts their frequency range [26]. The operating frequency of piezoelectric transmitters generally falls within the range of 0 kHz to 25 kHz, making them incapable of identifying fine-scale structures. Additionally, their low radiation energy and rapid attenuation during propagation limit long-distance transmission and detection, restricting their effective range to only a few dozen meters around the borehole.

The plasma emitter operates while immersed in a liquid. The application of high voltage and strong energy induces the breakdown of the gap within the emitter, forming a plasma channel and generating acoustic pulse effects that result in the instantaneous propagation of a powerful spherical pulse-wave. Compared to traditional acoustic wave detection sources, the plasma emitter has features such as a larger impulse wave amplitude, a wider frequency range, and the ability to be periodically triggered. In addition, it is possible to mount the plasma emitter and receiver array on the drilling instrument, allowing for detection without the need for sampling, thus avoiding environmental disturbance. The plasma emitter has significant application potential in the field of acoustic wave detection in areas such as petroleum geological exploration, marine seismic exploration, and oilfield deblocking [27,28].

With the continued development of high-voltage pulse-wave discharge technology for plasma emitters and the expanding range of applications, reactor structures for pulsed liquid electric discharge have become increasingly diverse [29,30]. Common reactor structures include rod–rod, needle–needle, and needle–plate structures. Among these, the needle–plate reactor structure is particularly prone to the formation of high-concentration plasma channels, which easily lead to excitation discharge phenomena [31]. In the early 20th century, Sunka and Suarasana [32,33] used multi-needle–plate discharge systems to treat drinking and industrial water and demonstrated that factors such as the number and density of needles, the gap length between the discharge point and the liquid surface, the thickness of the liquid layer, conductivity, and the discharge time affect the efficiency of ozone generation. In 2018, Wu Minggan, Liu Yi, and others [34,35], based on their research on a liquid electric impulse wave experimental platform, showed that the needle–plate structure can achieve high energy conversion efficiency and intense impulse wave intensity under dynamic breakdown conditions. Qian Yang et al. [36] developed a model of the needle–plate structure and demonstrated the formation of nearly cylindrical plasma channels between the needle and the plate.

To meet the application demands across various fields, researchers have made corresponding modifications and improvements to reactor structures. In 2023, Yin Kaiqiang [37] conducted an experimental study on the discharge characteristics of lightning rods, revealing that streamers are more likely to form on reactor structures with smaller curvature radii. In the same year, Wang Chen [38] experimentally investigated the impact of different curvature radii on emission spectrum characteristics, demonstrating that a smaller electrode tip curvature radius results in a higher electric field strength, which induces more intense discharge reactions and radiates greater energy. Additionally, Li Danhong [39] modified the rod structure into a hollow design, and modeling calculations indicated that the hollow rod generates a more intense non-uniform electric field, producing stronger impulse waves.

In order to investigate the pulse-wave sound pressure amplitude, frequency bandwidth, and electroacoustic energy conversion efficiency of the plasma emitter, we established a high-voltage discharge experiment for the plasma needle–plate emitter and developed a simulation model for discharge in the liquid medium. The experimental analysis focused on the discharge characteristics of the plasma needle–plate emitter. To meet the requirements of long-distance and high-precision acoustic signal transmission in downhole environments, structural advancements were made to the needle–plate emitter by reducing the curvature radius of the reactor structure and incorporating a hollow reactor design. This study proposes a hollow needle–plate emitter with a spherical tip, which is more advantageous for detecting and characterizing small-scale, deeply buried, and unconventional resources.

## 2. Methods

### 2.1. Principle

The generation and propagation of plasma emitter sources in liquids involves complex physical, chemical, and electrical processes that are easily influenced by factors such as reactor structure, circuit parameters, and the environmental conditions of the liquid. The generation and propagation of plasma emitter sources can be divided into three stages: the pre-breakdown phase, mid-breakdown phase, and post-breakdown phase.

The pre-breakdown phase is the initial stage of emission source generation. The needle–plate structure is immersed in tap water and the intense energy from the high-voltage capacitor is transferred to the needle–plate structure, causing a local electric field enhancement at the tip of the needle and the plate. This leads to the surrounding medium heating up, forming microbubbles and a breakdown ignition region. Water molecules in the vicinity of this region are heated, vaporized, and ionized. During the pre-breakdown phase, the initial stored energy of the capacitor can be calculated.

(1) $$E_{c}=CU_{0}^{2}/2$$

where *U*_0_ is the initial voltage of the capacitor and *C* is the capacitance value.

The mid-breakdown phase involves the formation and development of the plasma channel flow column between the needle–plate structures and the process of connecting the structural gaps until breakdown. When the electric field is present for a sufficient time, the vaporization and ionization cycles from the pre-breakdown phase form a flow column under a weaker field strength, which then develops towards the other end [40]. This forms an initial arc until the structural gap is broken, at which point a plasma channel is established between the structural gaps.

The post-breakdown phase refers to the process from the formation of the arc in the mid-breakdown phase to the generation of impulse waves and discharge currents. By continuously applying high field strength in the external circuit, the energy from the capacitor is rapidly injected into the plasma channel, accelerating the expansion of the arc and compressing the surrounding water medium. Due to the incompressibility of the medium, this results in the generation of strong impulse waves. During the intense discharge process, a modified gas state equation is used to describe the pressure *P* within the plasma channel.

(2) $$P=nkT-\frac{\mu_{0}I^{2}}{8\pi^{2}R_{ch}^{2}}-\frac{e^{2}}{32\pi^{2}\varepsilon_{0}}{\left(\frac{4\pi n}{3}\right)}^{4/3}$$

where $\mu_{0}=4\pi \times 10^{-7}H/m$ is the vacuum permeability, $e=1.6\times 10^{-19}C$ is the unit charge, $\varepsilon_{0},k,n$ are constants, *T* is the plasma channel temperature, and *I* is the channel current.

Therefore, the intensity of the impulse wave at distance *D* from the level of the center of the gap in the discharge structure is

(3) $$P_{D}=\frac{P\cdot b}{D}$$

where *b* is the radius of the plasma channel.

The expression for the impulse energy is given by [41]

(4) $$E_{w}=\frac{4\pi D^{2}}{\rho_{0}c_{s}}\int {P_{D}}^{2}dt$$

where $P_{D}$ is the intensity of the excitation wave, $\rho_{0}$ = 1000 $kg/m^{3}$ is the density of the water, and $c_{s}=1500m/s$ is the speed of sound in the water.

During the underwater plasma discharge process, there exists a certain degree of energy loss, and whether a high energy utilization rate can be obtained is also an issue we need to consider further. The conversion efficiency of the external circuit injecting energy to the impulse wave energy is defined as electroacoustic energy conversion efficiency $\eta_{0}$.

(5) $$\eta_{0}=\frac{E_{w}}{E_{c}}\times 100\%$$

**Figure 1:**
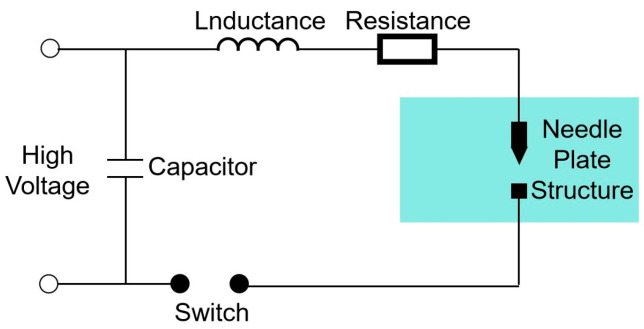
Equivalent circuit diagram of discharge circuit.

The equivalent circuit diagram of the external discharge loop of the plasma emission source is shown in Figure 1. *C* is the energy storage capacitance, *L* is the loop inductance, and *R* is the loop resistance; then, the differential equation of the equivalent loop is as follows:

(6) $$L\frac{di(t)}{dt}+\left(R+R_{ch}\right)i\left(t\right)-U_{C}\left(t\right)=0$$

where i(t) is the discharge current, $U_{C}(t)$ is the storage capacitor voltage, $R_{ch}$ is the plasma channel resistance, *R* is the loop resistance, and *L* is the loop inductance.

Since the equivalent circuit is an RLC circuit, it makes the whole circuit underdamped oscillations. The discharge current expression can be obtained by solving the above equivalent circuit differential Equation (6):

(7) $$i\left(t\right)=\frac{U_{C}}{\omega }e^{-t/\tau }sin(\omega t)$$

where $\tau =\frac{2L}{R_{ch}+R}$ and $\omega ={\left(\frac{1}{LC}-\frac{1}{\tau^{2}}\right)}^{1/2}$.

### 2.2. Experiment

**Figure 2:**
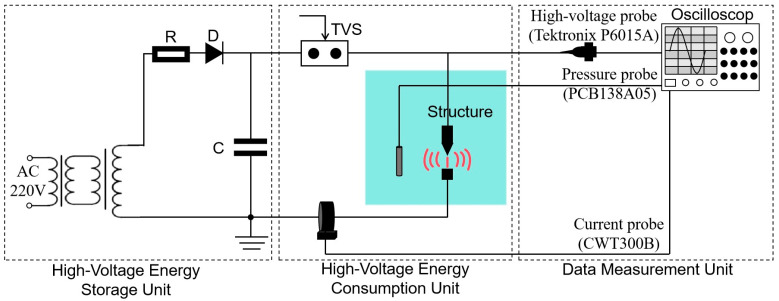
Schematic diagram of the experimental system for plasma emission source discharge in liquid.

**Figure 3:**
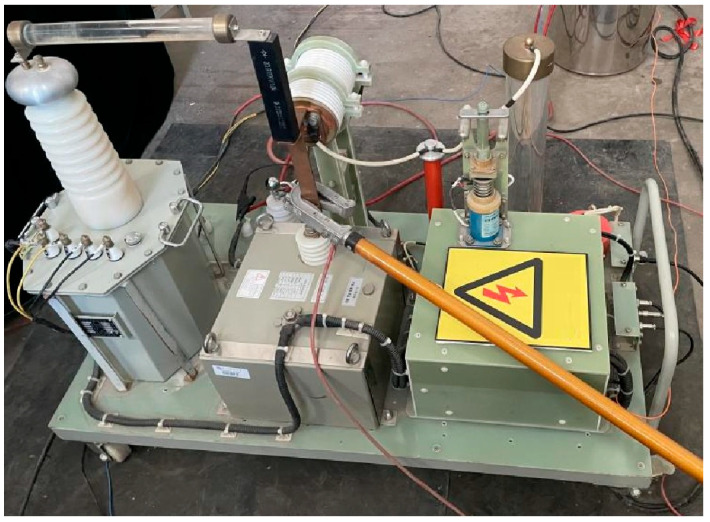
Physical diagram of the experimental system for plasma emission source discharge in liquid.

The principle of the liquid plasma emission source discharge experimental system is shown in Figure 2. It mainly consists of a high-voltage energy storage unit, a high-voltage energy consumption unit, and a data measurement unit. Figure 3 shows a physical diagram of the experimental system for plasma emission source discharge in liquid.

The high-voltage energy storage unit is constructed as follows: the regulator is connected to the step-up transformer, which is rectified by silicon stack D to charge the energy storage capacitor C with DC at high voltage. The high-voltage energy consumption unit is constructed as follows: when the charging voltage reaches a certain set value, the TVS (triggered vacuum switch) is triggered to make it conductive. Stored energy capacitor C is instantly applied to the needle–plate discharge structure and starts discharging. The needle–plate structure has a 5 mm gap and is placed in a stainless-steel bucket with tap water. The experimental charging voltage is 20 kV and the stored capacitance C is 15 μF. The data measurement unit is constructed as follows: A high-voltage probe (P6015A, Tektronix, Beaverton, OR, USA) and a current probe (CWT300B, Power Electronic Measurements Ltd., Long Eaton, UK) are used to measure the discharge voltage and current waveforms at the two ends of the needle–plate structure. A PCB pressure sensor (PCB138A05, PCB Piezotronics, Buffalo, NY, USA) is used to measure the excitation waveforms. The pressure probe is placed at a horizontal distance of 8 cm from the center of the gap in the needle–plate structure. An oscilloscope is used to synchronously record the voltage, current, and impulse waveforms of the discharge process.

### 2.3. Results

**Figure 4:**
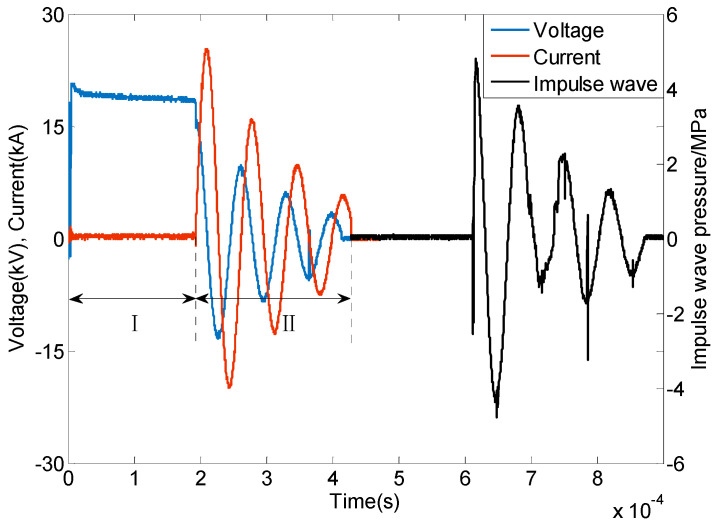
Typical voltage, current, and impulse waveforms for plasma needle–plate emission source discharge in liquids.

In our plasma needle–plate emission source discharge experiment in liquid, the voltage, current, and excitation wave of the discharge experiment process were measured by the high voltage probe, current probe, and pressure sensor, respectively. Typical voltages, currents, and excitations are shown in Figure 4.

As seen from the voltage waveform in Figure 4, phase I is the pre-breakdown and mid-breakdown phase (0~190 μs) of the plasma discharge in the liquid. Phase II is the post-breakdown phase (>190 μs). Due to the short duration of the breakdown process, it is not possible to obtain the specific pre-breakdown and mid-breakdown phases from the experimental results of the waveforms. As seen from the current waveform in Figure 4, at 15 μs after breakdown, the discharge current reaches the first peak value of 25 kA, and the discharge current and voltage show the second-order oscillation decay phenomenon, and the first peak value of the excitation wave actually measured by the PCB pressure probe is 5 MPa.

## 3. Enhancement

### 3.1. Validation

**Figure 5:**
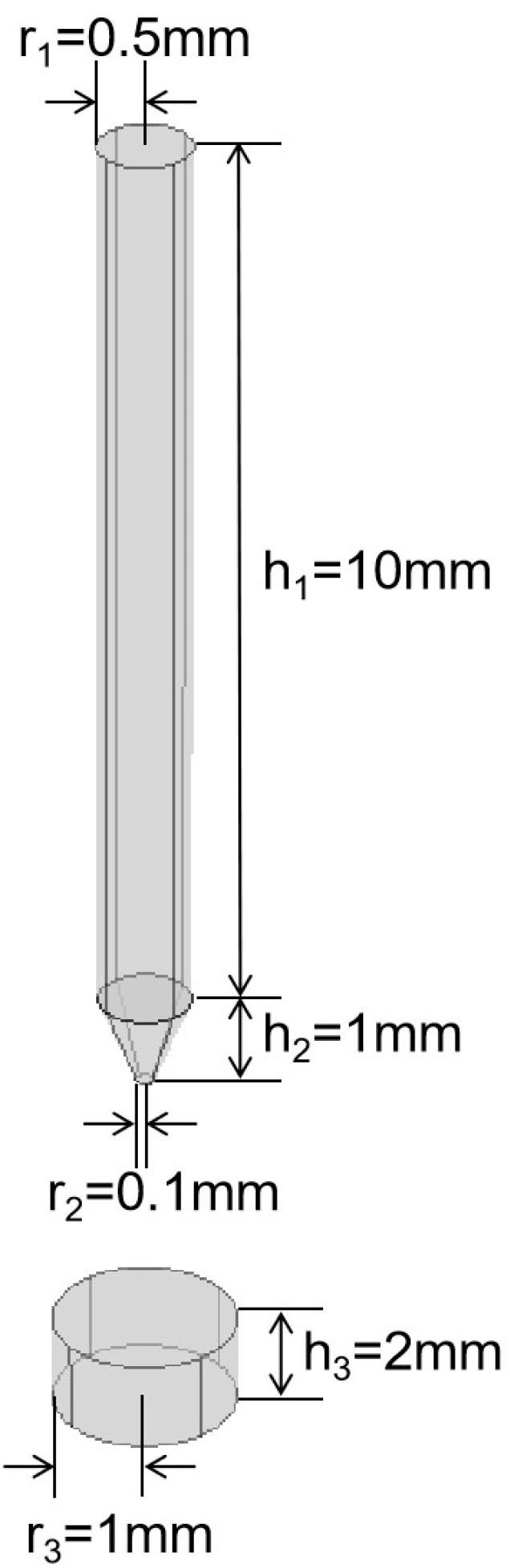
Plasma needle–plate emission source modeling diagram.

Modeling and numerical simulations were carried out using the multiphysics simulation software Comsol Multiphysics 5.6. A diagram of the plasma needle–plate emission source model is shown in Figure 5. The geometrical parameters of the plasma needle–plate emission source are given in Table 1. The anode is a stainless-steel needle of 10 mm length and 1 mm diameter. The cathode is a circular stainless-steel plate with a diameter of 2 mm and a thickness of 1 mm. The external circuit of the liquid plasma discharge is equivalent to an RLC circuit and the outputs of the circuit are connected to the anode and cathode, respectively. The 15 μF energy storage capacitor used in the experiment, combined with the experimental discharge current waveform, can be calculated as the equivalent inductance of the discharge circuit, which is 8 μH, with the equivalent resistance being 100 mΩ.

When performing the physical field setup, the water medium was set as the current field and fluid heat transfer field. A multiphysics coupling of electromagnetic heating was selected for transient simulation. The reference temperature was set to 273.15 K, and the ambient pressure was 101,325 Pa. Since the volume of the water tank is significantly larger than that of the electrodes and heating is concentrated, the side and bottom surfaces of the water tank were designed as thermally insulated boundary conditions. Additionally, to ensure electrical insulation of the model, the top surface of the water tank was defined as the water–air interface.

**Figure 6:**
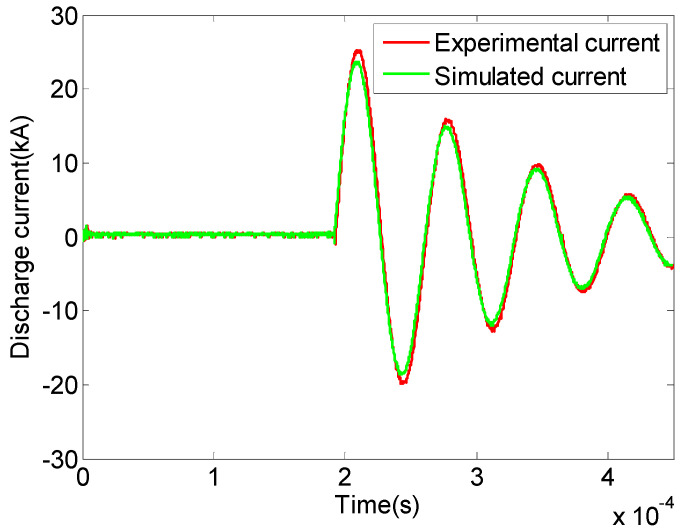
Measured and simulated discharge current of plasma needle–plate emitter in liquid.

The discharge current waveforms obtained from experimental measurements and numerical simulations of the plasma needle–plate emission source in liquid are shown in Figure 6. The measured and simulated electroacoustic energy conversion efficiencies can be calculated using Equations (1), (4), and (5). The comparative analysis of the simulated and measured results of the plasma needle–plate emission source in liquid is shown in Table 2.

By comparing and analyzing Figure 6 and Table 2, it can be seen that the simulated pre-breakdown time is shorter than the measured result because the measured result sums up the pre-breakdown and mid-breakdown together. The peak impulse intensity is lower than the measured result because the influence of external environmental factors such as light and acoustic radiation energy is neglected in the numerical simulation. The plasma channel is assumed to be an ideal columnar model in the simulation, resulting in a slight difference in the discharge current. The simulation results of discharge current, peak excitation, and electroacoustic energy conversion efficiency at the time of pre-breakdown are all in good agreement with the measured data.

### 3.2. Enhancement

As the curvature of the tip of the needle structure decreases, so does the inhomogeneity of the electric field. This inhomogeneous electric field can significantly reduce the pre-breakdown time and increase the excitation energy. Spheres have a smaller radius of curvature and higher charge density characteristics. Therefore, a sphere is used to locally optimize the needle–plate structure by adding a sphere to the tip of the needle structure. At the same time, the needle structure is changed to a hollow type so that it can generate a stronger inhomogeneous electric field during the discharge process.

**Figure 7:**
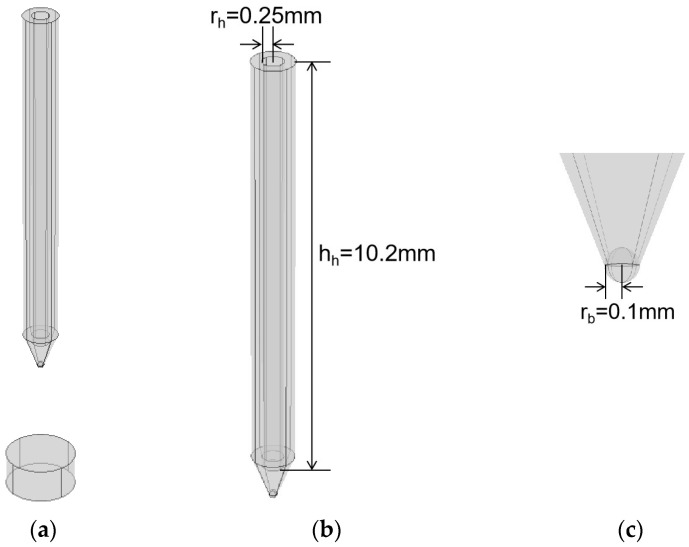
Model diagram of hollow needle–plate emitter with spherical tip: (**a**) overall diagram; (**b**) hollow needle structure; (**c**) spherical-tip needle structure.

A model diagram of the constructed plasma cylindrical spherical needle–plate emission source is shown in Figure 7. In this case, based on the needle–plate structure, a cylinder with a radius of 0.25 mm and a height of 10.20 mm was excavated to the center of the needle structure to form a hollow needle structure. A sphere with a radius of 0.1 mm was added to the tip of the needle structure to form a spherical-tip needle structure. The final result was a hollow needle–plate emitter with a spherical tip.

### 3.3. Results

**Figure 8:**
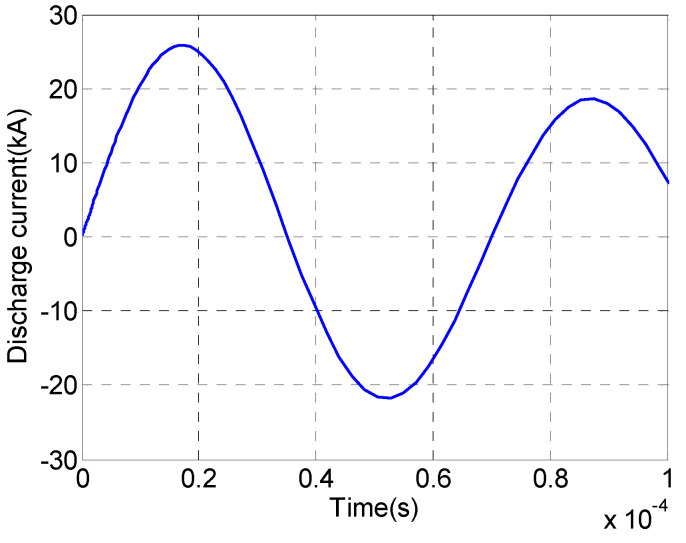
Discharge current waveform of the plasma hollow spherical-tip needle–plate emitter during the discharge process.

**Figure 9:**
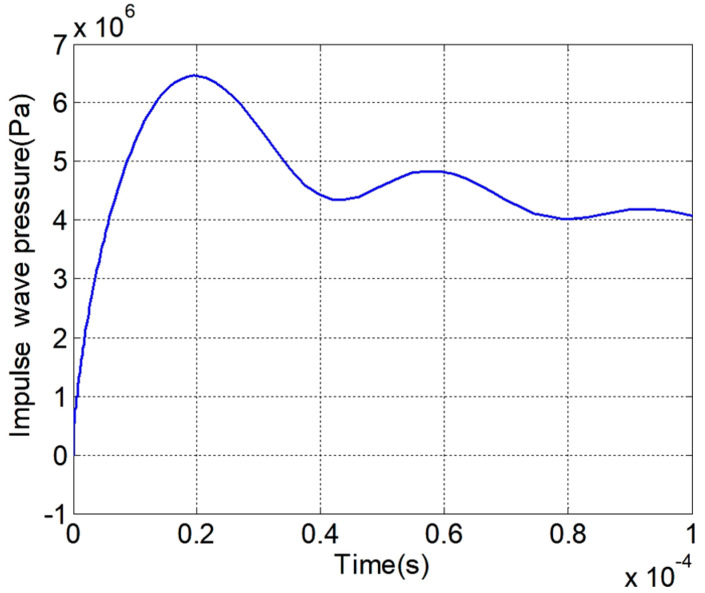
Impulse wave pressure versus time for the plasma hollow spherical-tip needle–plate emitter.

To model the plasma hollow spherical-tip needle–plate emitter, we used the same physical field settings and temperature–pressure settings as those used for the model validation in Section 3.1. We allowed the plasma hollow spherical-tip needle–plate emitter to fail. The plasma hollow spherical-tip needle–plate emitter was studied and analyzed in the simulation. The discharge current of the discharge process of the plasma hollow spherical-tip needle–plate emitter and the excitation pressure–time diagram of the plasma hollow spherical-tip needle–plate emitter are plotted in Figure 8 and Figure 9, respectively.

As shown in Figure 9, the maximum peak discharge current of the hollow spherical-tip needle–plate emitter during the discharge process is 25.77 kA, and the current gradually oscillates and decays thereafter. As shown in Figure 9, the maximum value of the impulse wave pressure is 6.46 MPa. Additionally, using Equations (1), (4), and (5), the electroacoustic conversion efficiency of the hollow spherical-tip needle–plate emitter is calculated to be 5.01%.

## 4. Discussion

After establishing the model for the hollow spherical-tip needle–plate plasma emitter, the same physical field parameters and temperature–pressure parameters used in the calculation of the needle–plate emitter are applied. The simulation model is then calculated, and high-voltage energy is applied to the hollow spherical-tip needle–plate emitter to induce a breakdown discharge. Equations (1), (4), and (5) are used to calculate the electroacoustic conversion efficiency of the externally injected energy into impulse wave energy. The results of the current, impulse wave pressure, and electroacoustic conversion efficiency of the hollow spherical-tip needle–plate emitter and the needle–plate emitter are summarized in Table 3.

As shown in Table 3, under the same environmental and model parameters, the needle structure of the needle–plate emitter was locally optimized. A spherical tip was added to the needle structure and a cylindrical cavity with a specific radius was excavated in the center of the needle structure. Through innovative improvements to the needle–plate emitter, the tip of the needle structure forms a highly fluctuating electric field, resulting in a significant amount of energy in the electric field. This facilitates the formation of a plasma channel between the needle structure and the plate structure. As a result, the peak discharge current of the hollow spherical tip needle–plate emitter is larger than that of the needle–plate emitter. The peak impulse wave pressure also increases from 5.08 MPa to 6.46 MPa, with a growth rate of 27.2%. The electroacoustic conversion efficiency also increases by 28.1%.

**Figure 10:**
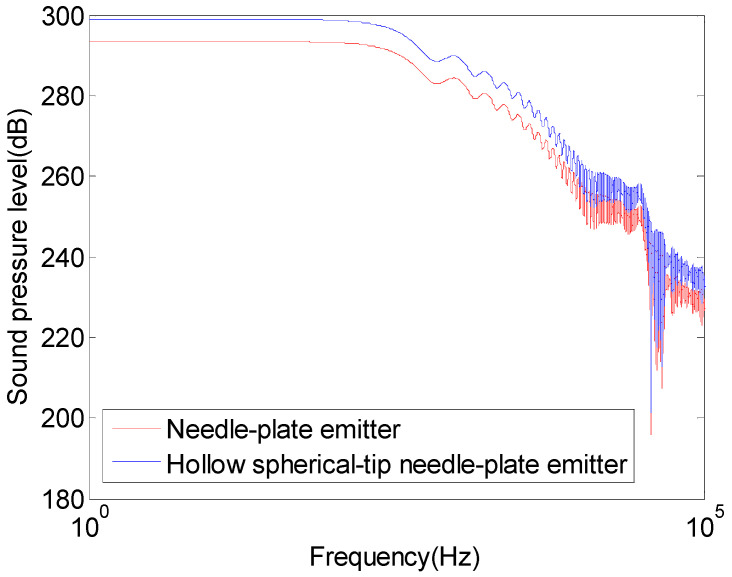
Sound pressure level–frequency graph of the hollow spherical-tip needle–plate emitter and the needle–plate emitter.

The impulse wave pressure of the hollow spherical-tip needle–plate emitter and the needle–plate emitter was converted into sound pressure level and underwent FFT transformation, as shown in Figure 10.

As shown in Figure 10, at the same frequency, the impulse sound pressure level of the hollow spherical-tip needle–plate is higher than that of the needle–plate emitter. When the excitation frequency is 0–1 kHz, the impulse wave sound pressure level of the hollow spherical-tip needle–plate emitter can reach 290 dB. When the excitation frequency is 1–10 kHz, the impulse wave sound pressure level can reach 260 dB. When the excitation frequency is 10–100 kHz, the impulse sound pressure level can reach 230 dB. By modifying the needle structure of the hollow spherical-tip needle–plate emitter to one that is hollow with a spherical tip, the impulse wave sound pressure level and energy can be increased, with an effective excitation frequency range of up to 100 kHz, enabling the creation of a three-dimensional acoustic field for structure detection within a hundred-meter range around the well. A comparison of the detection performance of the hollow needle–plate emitter with a spherical tip and conventional acoustic detection emitters is summarized in Table 4.

As shown in Table 4, monopole emitters, dipole emitters, phased-array emitters, and multipole emitters each have unique characteristics in terms of radiation frequency, radiation energy, detection range, resolution, and directionality, but these cannot be considered simultaneously. The plasma hollow spherical-tip needle–plate emitter, derived from the optimized structure of the needle–plate emitter, has superior detection performance. It has a wide radiation frequency band up to 100 kHz and has significant radiation energy. When surveying “deeper, thinner and smaller” target reservoirs and unconventional reservoirs, it can achieve long-range and high-precision acoustic transmission. The plasma hollow spherical tip needle–plate emitter, when excited with high sound energy at low radiation frequencies, can detect large structures such as oil–water interfaces and major fractures hundreds of meters away, enabling long-distance targeting for rescue wells and U-shaped wells. When excited with high sonic energy at high radiation frequencies, it can detect smaller structures such as residual oil, thin interlayers, and microfractures. When used downhole, it can accurately control wellbore trajectories, reducing the requirements for the inclination rate of the drilling equipment. This technology has significant application prospects in onshore and offshore oil and gas exploration, unconventional resource detection, and impulse wave fracturing and deblocking.

## 5. Conclusions

Based on traditional acoustic wave detection emitters, a plasma emitter was proposed. In order to investigate the impulse wave sound pressure amplitude, excitation frequency, and electroacoustic energy conversion efficiency of the impulse wave, high-voltage discharge experiments of the plasma needle–plate emitter were conducted, and a simulation model of the discharge of the emitter in liquid was developed. Considering the current demand for the long-distance and high-precision transmission of acoustic signals in wave detection, a new type of hollow needle–plate emitter with a spherical tip was proposed. The main conclusions are as follows:

(1) Traditional acoustic detection relies on piezoelectric emitters. The radiation frequency of these emitters is constrained by the limited downhole space, fixed within the range of 0 kHz to 25 kHz. Additionally, low radiation energy allows for detection only within a few tens of meters around the wellbore. This limitation makes them inadequate for future exploration of deeper reservoirs, smaller-scale formations, and more complex conventional and unconventional hydrocarbon reservoirs.
(2) A plasma emitter was proposed, and discharge experiments of the plasma needle–plate emitter in liquid were conducted. The needle–plate emitter achieved a conductive breakdown discharge in a water medium, with the first impulse wave peak measured by a PCB pressure sensor reaching 5 MPa.
(3) The structure of the needle–plate emitter was innovatively improved. A cylindrical cavity was introduced at the center of the needle structure, forming a hollow needle design. Additionally, a spherical tip was added to the needle’s tip, resulting in a spherical-tip needle structure. This optimization led to the development of a novel hollow spherical-tip needle–plate emitter. Compared with the needle–plate emitter, the impulse wave amplitude of the hollow spherical-tip needle–plate emitter increased by 27.2%, the electromechanical conversion efficiency improved by 28.1%, and the radiation frequency band can cover up to 100 kHz.
(4) The novel hollow spherical-tip needle–plate emitter enables long-range and high-precision acoustic wave transmission. It can detect small structures such as residual oil, thin interlayers and tiny fractures, as well as large structures such as oil–water–gas interfaces and large fractures at distances of hundreds of meters. It has enormous application potential in onshore and offshore oil and gas exploration and unconventional resource development. This study presents a preliminary investigation of the hollow spherical-tip needle–plate emitter. Future work will focus on a detailed examination of the effects of different hollow and spherical-tip structure sizes on the emitter’s performance, aiming to determine the optimal structural parameters. Additionally, a physical prototype of the hollow spherical-tip needle–plate emitter will be fabricated for discharge experiments.

## Figures and Tables

**Table 1:** The geometric parameters of the plasma needle–plate emitter.

Structural Part	Parameters
Needle tip radius/mm	0.1
Needle base radius/mm	0.5
Needle tip height/mm	1
Needle shaft height/mm	10
Plate structure radius/mm	1
Plate structure height/mm	1

**Table 2:** Comparison and analysis of simulated and measured results of plasma needle–plate emitter in liquid.

	Measured Results	Simulated Results	Relative Error
Pre-breakdown phase time/ms	0.19	0.181	−4.74%
Peak discharge current/kA	25	25.18	0.72%
Peak impulse wave intensity/MPa	5	5.08	1.60%
Electroacoustic energy conversion efficiency	3.91	3.95	1.02%

**Table 3:** Comparative analysis of the results for the hollow spherical-tip needle–plate emitter and the needle–plate emitter.

	Peak Discharge Current (kA)	Maximum Impulse Wave Pressure (MPa)	Electroacoustic Conversion Efficiency (%)
Needle–Plate Emitter Source	25.18	5.08	3.91
Hollow Spherical Tip Needle–Plate Emitter Source	25.77	6.46	5.01
Growth Rate	2.3%	27.2%	28.1%

**Table 4:** Comparison of detection performance of emitters.

Emitter Source	Radiation Frequency	Propagation Attenuation	Detection Range	Detection Resolution	Directional Directivity
Monopole Emitter [42]	10–15 kHz	Fast	A few meters	Good	Poor
Dipole Emitter [43]	Below 5 kHz	Medium speed	Tens of meters	Poor	Moderate
Phased-Array Emitter [44]	14 kHz	Fast	Tens of meters	Moderate	Good
Multipole Emitter [45]	15–25 kHz	Slow	Tens of meters	Good	Moderate
Hollow Spherical-Tip Needle–Plate Emitter	Below 100 kHz	Dependent on frequency, adjustable	Hundreds of meters	Dependent on frequency, adjustable	Good (with focusing device)

## Data Availability

The original contributions presented in the study are included in the article; further inquiries can be directed to the corresponding author.
